# Effect of acute dietary nitrate intake on post-activation performance enhancement in male long jumpers

**DOI:** 10.3389/fnut.2026.1823053

**Published:** 2026-06-25

**Authors:** Zhanming Xu, Jiawei Sun, Shiyi Xu, Jianing Gu, Shuwen Jiang, Jing Guo, Ju Lin, Laikang Yu

**Affiliations:** 1 College of Education, Beijing Sport University, Beijing, China; 2 Key Laboratory of Sport Training of General Administration of Sport of China, General Administration of Sport, Beijing, China; 3 School of Competitive Sports, Beijing Sport University, Beijing, China; 4 Tongliao No.5 Senior High School, Tongliao, China; 5 Chengdu Shishi United Middle School Shuhua Branch, Chengdu, China; 6 Beijing Key Laboratory of Sports Performance and Skill Assessment, Beijing Sport University, Beijing, China; 7 Department of Strength and Conditioning Assessment and Monitoring, Beijing Sport University, Beijing, China

**Keywords:** beetroot juice, countermovement jump, dietary nitrate, long jumper, post- activation performance enhancement, velocity loss

## Abstract

**Background:**

This study aimed to determine whether acute dietary nitrate supplementation enhances the post-activation performance enhancement (PAPE) response induced by squats performed with a 5% velocity loss (VL) threshold.

**Methods:**

A randomized, double-blind, placebo-controlled, crossover design was employed. 20 male long jumpers completed three experimental conditions: placebo (PLA), 5%VL, and beetroot juice (BRJ) + 5%VL. Approximately 2 h following the ingestion of 100 ml of BRJ containing 9.22 mmol of nitrate or a nitrate-depleted placebo, participants performed a squat conditioning protocol at 85% their one-repetition maximum (1RM), employing a 5%VL threshold. Countermovement jump (CMJ) performance was assessed at baseline and at 4, 8, 12, and 16 min post-intervention.

**Results:**

At 4 min post-intervention, all three groups showed significant improvements from baseline in CMJ height, peak power output (PPO), and momentum (*P* < 0.05), with no significant differences observed between the groups. The total number of squat repetitions performed across two sets was significantly higher in the BRJ + 5%VL group compared to both the 5%VL (*P* = 0.002) and PLA groups (*P* = 0.027).

**Conclusion:**

Squats performed with a 5% VL threshold effectively elicited a PAPE response in male long jumpers, resulting in improved short-term explosive performance. In contrast, acute BRJ supplementation did not enhance the PAPE response or further improve single-jump performance. However, BRJ supplementation was associated with a greater number of squat repetitions completed at 85%1RM, suggesting a potential benefit for squat repetition tolerance and strength-endurance capacity. These findings indicate that low-volume activation protocols may be useful for acutely enhancing explosive performance, whereas acute dietary nitrate supplementation appears unlikely to augment PAPE but may contribute to improved resistance exercise tolerance.

## 1 Introduction

In recent years, sport science research has focused on strategies capable of acutely enhancing athletic performance prior to competition or training. These strategies generally encompass physical preparation approaches and nutritional interventions (1–3). Among physical preparation methods, the induction of post-activation performance enhancement (PAPE) through short-duration, high-intensity exercise has emerged as a widely investigated topic (4, 5). PAPE refers to a transient enhancement of neuromuscular function following a conditioning activity, whereby subsequent explosive performance may be improved if an appropriate recovery interval is implemented (6). Although the existence of PAPE has been extensively documented (7–9), its manifestation depends on specific eliciting conditions, typically requiring moderate-to-high or near-maximal intensity stimulation. Moreover, its ergogenic benefits are predominantly observed in short-duration, high-intensity explosive tasks. Notably, Yuan et al. (10) reported peak PAPE effects at 8 min post-conditioning, whereas Sun et al. (11) observed significant improvements as early as 4 min. These discrepant temporal responses underscore the importance of assessing multiple recovery time points to capture individualized PAPE dynamics.

Strength training is commonly employed to induce PAPE (12, 13). However, traditional load prescription based on a percentage of one-repetition maximum (%1RM) presents several limitations (14), including procedural complexity, the need for maximal strength testing, and an increased potential risk of injury. In contrast, velocity-based training (VBT) has recently gained prominence as a more precise and individualized approach for optimizing PAPE protocols, owing to its capacity for real-time load monitoring and fatigue regulation (15, 16).

From a nutritional perspective, the International Olympic Committee has identified several evidence-based ergogenic supplements for high-intensity performance, including caffeine, creatine, and dietary nitrate (17). Among these, dietary nitrate, commonly derived from beetroot and other nitrate-rich vegetables (18), has received growing attention as a plant-based performance-enhancing intervention (19). The primary mechanism underlying dietary nitrate supplementation involves its sequential reduction to nitric oxide (NO), a bioactive molecule that influences multiple physiological systems. Nitric oxide has been shown to improve muscle contractile efficiency (20, 21), reduce the oxygen cost of exercise (22), enhance local blood flow (23), and exert preferential effects on fast-twitch, fatigue-prone type II muscle fibers (23–25). Collectively, these mechanisms mitigate fatigue accumulation during repeated high-intensity contractions, thereby increasing the capacity to perform a greater number of repetitions at submaximal loads—a key determinant of strength-endurance performance. Emerging evidence suggests that nitrate supplementation may enhance skeletal muscle power output (20, 21, 26), particularly in type II fibers (21), potentially through modulation of calcium release or increased calcium sensitivity, although the precise molecular pathways remain incompletely understood (20, 27).

Accumulating research indicates that dietary nitrate supplementation can improve performance in intermittent high-intensity exercise (28), endurance exercise (29), and certain resistance training modalities (30); however, its ergogenic effects appear to be context-dependent. Notably, benefits are more consistently observed in exercise modes characterized by higher contraction velocities and greater type II fiber recruitment (e.g., high cadence cycling) (24, 25, 31). Furthermore, its performance-enhancing effects appear to be most pronounced during the initial phase of muscle contraction. For example, improvements in very short-duration sprint performance (e.g., ~6 s) tend to exceed those observed in longer-duration efforts (e.g., ~30 s) (32). Recent meta-analyses similarly report that nitrate supplementation is more likely to enhance peak power during single maximal sprints rather than repeated sprint performance (33). Collectively, these findings suggest that dietary nitrate may preferentially optimize rapid, high-power muscle contractions dominated by type II fiber activity. This study employed a concentrated beetroot juice (BRJ) supplement (Beet It Sport^®^) delivering 9.22 mmol of nitrate per 100 ml, a dosage consistent with levels previously shown to enhance high-intensity performance (33).

Given these characteristics, a compelling hypothesis is that acute dietary nitrate supplementation may interact synergistically with PAPE protocols designed to transiently enhance neuromuscular performance. PAPE typically involves the use of submaximal or maximal conditioning loads, such as heavy squats, to acutely improve subsequent explosive tasks (34). Theoretically, nitrate supplementation could enhance the quality of the conditioning activity itself by improving contraction efficiency and neural drive, thereby generating a more potent activation stimulus. Additionally, its facilitation of early phase contractile function may amplify the performance enhancement window induced by PAPE. Such a combined intervention model, integrating nutritional supplementation with a targeted training stimulus, may produce superior performance outcomes compared with either strategy applied independently.

Previous systematic reviews and original investigations have examined the acute effects of co-administering caffeine, creatine, or carbohydrate mouth rinse with conditioning activities to potentiate PAPE responses (35, 36). Despite these theoretical considerations, no previous studies have directly examined whether acute dietary nitrate supplementation can augment the magnitude of the PAPE effect. The interaction between these two performance-enhancing strategies therefore represents a notable gap in the literature. Accordingly, the present study aimed to investigate whether acute dietary nitrate intake potentiates the PAPE response induced by squats performed at a 5%VL threshold in male long jumpers. We hypothesized that, compared with placebo, acute dietary nitrate supplementation would significantly enhance the PAPE effect elicited by the conditioning activity, as reflected by improved neuromuscular performance and an increased number of squat repetitions at a fixed VL threshold during subsequent testing.

## 2 Materials and methods

### 2.1 Participants

Sample size was calculated using G^*^Power (repeated-measures ANOVA; effect size *f* = 0.25, α = 0.05, power = 0.95), indicating a minimum of 15 participants. Considering a potential attrition rate of approximately 20%, 20 male long jumpers from Beijing Sport University were ultimately recruited. Athlete eligibility was determined according to McKay's training level classification (37), and Tier 2 athletes were selected to ensure a homogeneous training background.

Inclusion criteria required participants to be free from cardiovascular or respiratory disease, to have no lower-limb joint injuries or neuromuscular disorders affecting performance within the previous 6 months, to be in good general health at the time of testing, and to possess structured resistance training experience, particularly in the barbell back squat. Exclusion criteria included: (1) known allergy to beetroot or related products; (2) chronic use of medications known to influence nitric oxide metabolism or vascular function (e.g., organic nitrates or phosphodiesterase inhibitors); and (3) regular consumption of nitrate-containing supplements or other ergogenic aids within 1 month prior to the study without willingness to discontinue use during the washout period.

All participants volunteered during the off-season and provided written informed consent after receiving a detailed explanation of the study purpose, procedures, potential risks, and benefits. The study protocol was approved by the Ethics Committee of Beijing Sport University (Approval No.: 2025586H) and conducted in accordance with the Declaration of Helsinki. Participant characteristics are summarized in Table 1.

**Table 1:** Physical characteristics of subjects at baseline (*n* = 20).

Variables	Mean ±SD
Age (years)	20.58 ± 1.36
Height (cm)	180.74 ± 4.98
Body mass (kg)	73.90 ± 3.76
Resistance training experience (years)	3.95 ± 1.00
1RM (kg)	159.47 ± 13.87

RM, repetition maximum.

### 2.2 Study design

A randomized, double-blind, placebo-controlled, crossover design was employed. Each participant completed three experimental sessions separated by washout intervals of 1–2 weeks to minimize carryover effects. To reduce variability in baseline nitrate status, participants were instructed to avoid nitrate-rich foods for one week prior to the first trial and throughout the study period. Dietary intake was recorded from the beginning of the first washout phase until completion of the final testing session, and compliance was verified by the research team. Participants were additionally instructed to refrain from using antibacterial mouthwash, brushing teeth, or chewing gum on testing days to avoid disruption of the oral nitrate-nitrite conversion pathway (31).

Participants arrived at the laboratory 3 h prior to testing and were randomly assigned to ingest either 100 ml of a commercially available concentrated BRJ supplement (Beet It Sport^®^, James White Drinks, Ipswich, UK; providing 9.22 mmol of nitrate per 100 ml) or an equivalent volume of a nitrate-depleted, taste- and appearance-matched placebo (ECO Saludviva, Alicante, Spain; nitrate-depleted via ion exchange resin). This dosage was selected as it lies within the range previously demonstrated to enhance muscle contractile function and resistance exercise performance, while remaining practical for acute supplementation protocols (38). Randomization was performed using Research Randomizer software (www.randomizer.org) by a researcher not involved in data collection to maintain allocation concealment (39). The timing of testing was standardized to coincide with peak plasma nitrate concentrations, approximately 2 h post-ingestion, based on prior literature (40–43).

Participants completed three experimental conditions in a randomized crossover design: (1) Control (5%VL), consisting of the 5%VL squat conditioning protocol without any supplementation; (2) Placebo (PLA), involving ingestion of the nitrate-depleted placebo beverage followed by the 5%VL squat conditioning protocol; and (3) BRJ + 5%VL, involving ingestion of the active BRJ supplement followed by the same 5%VL squat conditioning protocol. Accordingly, the primary comparison of nitrate-specific effects was made between the PLA and BRJ + 5%VL conditions, whereas the 5%VL condition served as a reference condition to account for any potential non-nitrate effects associated with placebo ingestion.

### 2.3 Procedures

#### 2.3.1 1RM squat test

The 1RM of the free-weight barbell back squat was determined using a validated protocol. Height and body mass were recorded prior to testing. Participants first completed a familiarization session with the VBT system to ensure correct execution of the movement and appropriate use of the velocity monitoring equipment. A standardized warm-up consisting of dynamic stretching and bodyweight exercises was then performed to optimize joint mobility and neuromuscular readiness. The 1RM test began with a 40-kg load and followed a progressive incremental protocol. When mean concentric velocity decreased below 0.5 m/s, the load was increased in 20-kg increments. As the 0.5 m/s threshold was approached, smaller individualized increments (1–5 kg) were applied to precisely determine 1RM.

All testing procedures adhered to NSCA technical standards (44). Participants positioned their feet approximately shoulder-width apart, with the barbell placed across the upper trapezius and the torso maintained upright. During the descent phase, the knees tracked in line with the toes, and squat depth was standardized to parallel (thighs parallel to the floor). During ascent, trunk stability was maintained throughout. Attempts were categorized according to concentric velocity: light (>0.7 m/s), moderate (0.7–0.5 m/s), and heavy (< 0.5 m/s), permitting three, two, and one attempt(s), respectively. The heaviest load successfully lifted once with proper technique was recorded as the 1RM. Participants were instructed to control the eccentric phase and perform the concentric phase with maximal intended velocity. Two experienced spotters supervised all trials to ensure safety. Rest intervals were standardized at 4 min for light and moderate attempts and 6 min for heavy attempts.

#### 2.3.2 PAPE protocol

A validated PAPE protocol was implemented. Following a standardized warm-up (Table 2) (45), participants rested for 5 min and then performed three countermovement jump (CMJ) trials to establish baseline values, with the highest jump height, peak power output (PPO), and takeoff momentum retained for analysis. After a 3-min rest, participants completed the conditioning activity consisting of two sets of squats at 85% 1RM (46), with a 1-min inter-set rest interval (47, 48). To standardize technique, a 5-cm-wide marker line was placed 30 cm behind the barbell. Participants stood with their feet aligned along this line, shoulder-width or slightly wider, and toes slightly externally rotated. A 1-m elastic band was suspended 50–70 cm above the marker line and adjusted to correspond with each participant's torso position at parallel squat depth to ensure consistent movement amplitude.

**Table 2:** Warm-up programs for PAPE protocol.

Warm-up component	Protocol details (for 5%VL, placebo and BRJ + 5%VL groups)
Jogging	5 min
Muscle activation	Shoulder circles: 1 set × 12 reps Mountain climber: 1 set × 18 reps Single leg glute bridges: 1 set × 12 reps Superman: 1 set × 8 reps Squat: 1 set × 8 reps
Dynamic stretching	Inchworm: 1 set × 8 reps 90/90 for hips: 1 set × 8 reps Lunge walk: 1 set × 8 reps Walking knee lift: 1 set × 8 reps
Neuromuscular activation	Split squat jump: 1 set × 5 reps Squat jump: 1 set × 5 reps Box jump: 1 set × 5 reps

Barbell velocity was monitored in real time using a GymAware Power tool linear position transducer (Kinetic Performance, Australia), positioned on the ground to the right of the squat rack and attached vertically to the barbell. Participants received standardized verbal encouragement and real-time feedback on mean concentric velocity. The VL threshold was calculated as (14):


$$\begin{array}{l} \text{Terminal velocity}=\text{Initial velocity }\times \;\left(1-\text{VL}\%\right). \end{array}$$

Where initial velocity corresponded to the first repetition of each set. Each set was terminated when repetition velocity fell below the predetermined threshold (49). This protocol was selected based on evidence indicating that high relative loads (≥ 85% 1RM) and multiple-set configurations can effectively induce PAPE, while a 1-min inter-set interval has been shown to optimize the balance between potentiation and fatigue.

Following the conditioning activity, CMJ assessments were conducted at 4, 8, 12, and 16 min post-intervention to examine the temporal characteristics of the PAPE response.

#### 2.3.3 Countermovement jump (CMJ)

CMJ testing followed a standardized and validated protocol. At each time point, participants performed three maximal CMJ trials with a 10-s inter-trial rest interval to minimize fatigue (10). Participants stood upright at the center of a three-dimensional force platform, with hands placed on hips and feet shoulder-width apart (50). Upon a standardized verbal cue, participants performed a rapid countermovement to a self-selected depth and immediately executed a maximal vertical jump. During flight, full knee extension was required and hands remained on the hips to eliminate arm swing influence.

The highest jump height of the three trials was retained for analysis. Jump height, peak power output, and takeoff momentum were recorded using a force platform (Kistler 9281CA, Switzerland; sampling frequency: 1,000 Hz) (51). All testing sessions were conducted under consistent verbal encouragement, and laboratory ambient temperature was maintained between 20 and 24°C (10).

### 2.4 Statistical analysis

All data are presented as mean ± standard deviation (SD). A two-way repeated-measures ANOVA (condition × time) was performed to examine main effects and interaction effects. Assumptions of sphericity were assessed using Mauchly's test; when violated, degrees of freedom were corrected using the Greenhouse–Geisser adjustment. For one-way repeated-measures ANOVA, sphericity was similarly verified and corrected using Greenhouse–Geisser or Huynh–Feldt adjustments where appropriate. Statistical significance was set at *P* < 0.05. Effect sizes were calculated as Cohen's d and interpreted as trivial (0–0.2), small (0.2–0.6), moderate (0.6–1.2), large (1.2–2.0), or very large (>2.0) (52). All statistical analyses were conducted using SPSS (version 25.0, IBM, Chicago, IL, USA), and graphical representations were generated using GraphPad Prism (version 8.3.0, GraphPad Software, San Diego, CA, USA).

## 3 Results

### 3.1 Effects of 5% VL squat PAPE intervention on CMJ height

Two-way repeated-measures ANOVA revealed no significant condition × time interaction for CMJ height (*F*_(8, 152)_ = 0.088, *P* = 0.999). There was no significant main effect of condition (*F*_(2, 38)_ = 1.713, *P* = 0.182), whereas a significant main effect of time was observed (*F*_(4, 76)_ = 3.886, *P* = 0.004), indicating a transient change in performance across post-intervention time points irrespective of supplementation.

**Figure 1:**
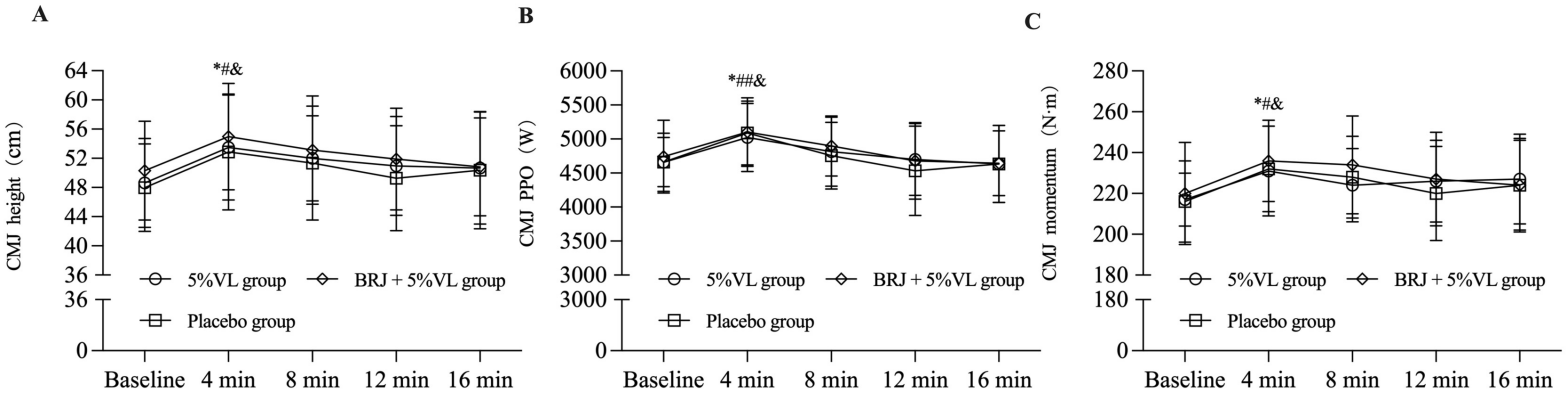
CMJ height **(A)**, PPO **(B)**, and momentum **(C)** during PAPE condition of three groups (*n* = 20). Compared with baseline, ^*^P < 0.05 (5%VL group), ^&^*P* < 0.05 (BRJ + 5%VL group), ^#^*P* < 0.05 (Placebo group), ^*##*^*P* < 0.01 (Placebo group). VL, velocity loss; BRJ, beetroot juice; PPO, peak power output.

As presented in Tables 3, 4 and Figure 1A, *post hoc* analyses revealed that CMJ height significantly increased at 4 min following the squat conditioning activity in all three conditions. Specifically, significant improvements were observed in the placebo condition (*P* = 0.035, Cohen's *d* = 0.693), the 5%VL condition (*P* = 0.027, Cohen's *d* = 0.725), and the BRJ + 5%VL condition (*P* = 0.043, Cohen's *d* = 0.661).

**Table 3:** Jump height, PPO and momentum of CMJ during PAPE condition of different groups (*n* = 20).

Sports performance variables	Group	Assessment time point				
		Baseline	4 min	8 min	12 min	16 min
CMJ height (cm)	Placebo group	47.99 ± 6.00	52.88 ± 7.96^#^	51.34 ± 7.83	49.28 ± 7.21	50.37 ± 8.05
	5%VL group	48.62 ± 6.11	53.47 ± 7.22^*^	51.99 ± 5.84	50.97 ± 6.79	50.66 ± 7.70
	BRJ + 5%VL group	50.31 ± 6.78	54.97 ± 7.30^&^	53.12 ± 7.44	51.88 ± 6.98	50.83 ± 6.71
CMJ PPO (W)	Placebo group	4660.78 ± 361.98	5088.10 ± 468.56^##^	4756.30 ± 492.26	4532.51 ± 655.68	4631.87 ± 568.64
	5%VL group	4658.14 ± 427.94	5024.34 ± 502.20^*^	4814.21 ± 506.57	4700.11 ± 527.66	4631.87 ± 568.64
	BRJ + 5%VL group	4739.50 ± 534.54	5102.53 ± 503.64^&^	4897.94 ± 440.73	4679.00 ± 561.01	4642.72 ± 475.86
CMJ momentum (Nm)	Placebo group	215.90 ± 19.52	231.86 ± 20.98^#^	227.52 ± 19.98	220.18 ± 23.41	224.11 ± 22.82
	5%VL group	216.67 ± 13.35	230.51 ± 21.77^*^	223.50 ± 17.92	226.25 ± 19.67	226.85 ± 21.57
	BRJ + 5%VL group	219.90 ± 25.41	235.68 ± 20.04^&^	233.85 ± 23.73	226.94 ± 22.55	224.25 ± 22.25

Compared with baseline, ^*^P < 0.05, ^&^P < 0.05, ^#^P < 0.05, ^##^P < 0.01. PAPE, post-activation performance enhancement; CMJ, countermovement jump; VL, velocity loss; BRJ, beetroot juice.

**Table 4:** Descriptive statistics of the acute effects of different variables before and after three group interventions (*n* = 20).

Sports performance variables	Group	Cohen's d with 95%CI			
		4 min	8 min	12 min	16 min
CMJ height (cm)	Placebo group	0.693 (0.055–1.331)	0.479 (−0.15–1.107)	0.193 (−0.428–0.814)	0.335 (−0.29–0.959)
	5%VL group	0.725 (0.085–1.365)	0.565 (−0.067–1.197)	0.363 (−0.262–0.988)	0.293 (−0.33–0.916)
	BRJ + 5%VL group	0.661 (0.024–1.297)	0.394 (−0.232–1.02)	0.228 (−0.394–0.85)	0.076 (−0.544–0.696)
CMJ PPO (W)	Placebo group	1.021 (0.362–1.68)	0.221 (−0.401–0.843)	−0.242 (−0.864–0.38)	−0.061 (−0.681–−0.559)
	5%VL group	0.785 (0.142–1.428)	0.333 (−0.291–0.957)	0.087 (−0.533–0.707)	−0.052 (−0.672–0.568)
	BRJ + 5%VL group	0.699 (0.061–1.337)	0.323 (−0.3–0.947)	−0.11 (−0.731–0.51)	−0.191 (−0.812–0.43)
CMJ momentum (Nm)	Placebo group	0.788 (0.144–1.431)	0.589 (−0.044–1.222)	0.199 (−0.423–0.82)	0.387 (−0.239–1.012)
	5%VL group	0.766 (0.124–1.409)	0.432 (−0.195–1.059)	0.57 (−0.062–1.203)	0.568 (−0.065–1.2)
	BRJ + 5%VL group	0.707 (0.068–1.346)	0.571 (−0.061–1.204)	0.291 (−0.332–0.915)	0.17 (−0.451–0.791)

CMJ, countermovement jump; VL, velocity loss; BRJ, beetroot juice.

### 3.2 Effects of 5% VL squat PAPE intervention on CMJ PPO

For CMJ PPO, no significant condition × time interaction was identified (*F*_(8, 152)_ = 0.183, *P* = 0.993), and no significant main effect of condition was found (*F*_(2, 38)_ = 0.596, *P* = 0.5523). However, a significant main effect of time was detected (*F*_(4, 76)_ = 7.848, *P* = 0.001), suggesting time-dependent alterations in PPO following the PAPE protocol.

As shown in Tables 3, 4 and Figure 1B, significant increases in CMJ PPO were observed at 4 min post-intervention in all three conditions. Improvements were evident in the placebo condition (*P* = 0.003, Cohen's *d* = 1.021), the 5%VL condition (*P* = 0.018, Cohen's *d* = 0.785), and the BRJ + 5%VL condition (*P* = 0.033, Cohen's *d* = 0.699).

### 3.3 Effects of 5% VL squat PAPE intervention on CMJ momentum

Two-way repeated-measures ANOVA revealed no significant condition × time interaction for CMJ momentum (*F*_(8, 152)_ = 0.312, *P* = 0.961), and no significant main effect of condition (*F*_(2, 38)_ = 1.368, *P* = 0.256). In contrast, a significant main effect of time was observed (*F*_(4, 76)_ = 4.033, *P* = 0.003), indicating that CMJ momentum varied across time points independent of supplementation condition.

As reported in Tables 3, 4 and Figure 1C, significant increases in CMJ momentum were found at 4 min post-conditioning in the placebo condition (*P* = 0.017, Cohen's *d* = 0.788), the 5%VL condition (*P* = 0.020, Cohen's *d* = 0.766), and the BRJ + 5%VL condition (*P* = 0.036, Cohen's *d* = 0.707).

### 3.4 Number of squat repetitions

**Figure 2:**
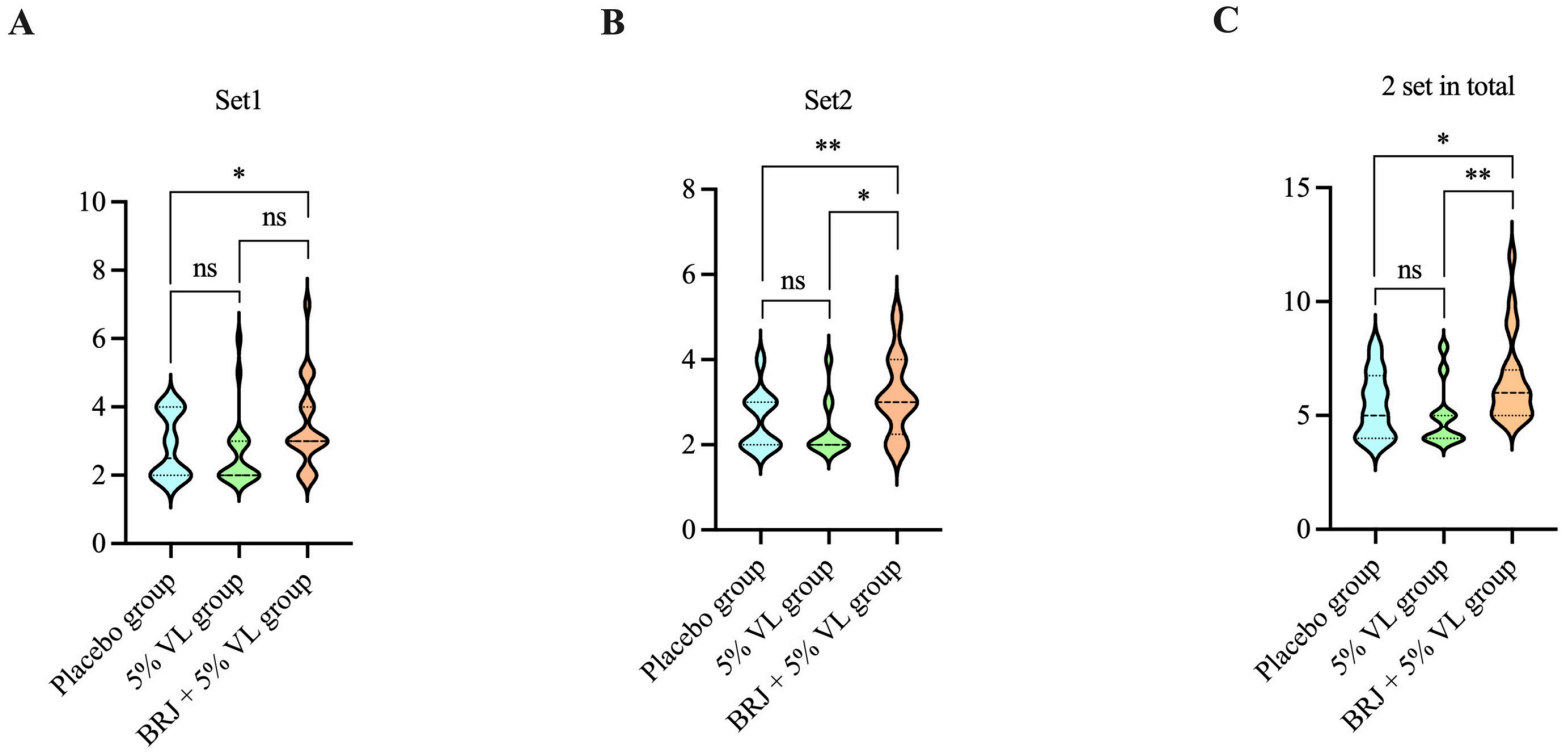
Comparisons of squat repetitions across placebo, 5%VL, and beetroot juice + 5%VL groups in set 1 **(A)**, set 2 **(B)**, and combined two sets **(C)**. ^*^P < 0.05, ^**^P < 0.01. VL, velocity loss; BRJ, beetroot juice.

For Set 1, one-way repeated-measures ANOVA indicated no statistically significant difference among conditions (*F*_(2, 38)_ = 2.821, *P* = 0.068). However, pairwise comparisons revealed that the number of repetitions performed under the 5%VL condition was significantly lower than that in the BRJ + 5%VL condition (*P* = 0.026, Table 5 and Figure 2A).

**Table 5:** Statistical table of squat repetitions for three sets under 5% VL (*n* = 20).

Group	Number of repetitions		
	Set 1 repetitions	Set 2 repetitions	Total repetitions (2 sets)
Placebo group	2.85 ± 0.93^a^	2.60 ± 0.68^a^	5.45 ± 1.4^a^
5%VL group	2.65 ± 1.09	2.35 ± 0.67	5.00 ± 1.38
BRJ + 5%VL group	3.45 ± 1.28^a^	3.15 ± 0.93^aab^	6.60 ± 1.96^aab^

Compared with 5%VL group, ^a^P < 0.05, ^aa^P < 0.01. Compared with PLA group, ^b^P < 0.05. VL, velocity loss; BRJ, beetroot juice.

For Set 2, a significant main effect of condition was observed (*F*_(2, 38)_ = 5.633, *P* = 0.006). *Post hoc* analysis demonstrated that the number of repetitions completed in the 5%VL condition was significantly lower than in the BRJ + 5%VL condition (*P* = 0.002), and the placebo condition was also significantly lower than the BRJ + 5%VL condition (*P* = 0.028, Table 5 and Figure 2B).

When total repetitions across both sets were analyzed, a significant effect of condition was identified (*F*_(2, 38)_ = 5.325, *P* = 0.008). Specifically, the total number of squat repetitions performed in the 5%VL condition was significantly lower than in the BRJ + 5%VL condition (*P* = 0.002), and the placebo condition was also significantly lower than the BRJ + 5%VL condition (*P* = 0.027, Table 5 and Figure 2C). These findings indicate that acute BRJ supplementation enabled participants to complete a greater training volume under the prescribed velocity loss threshold.

## 4 Discussion

### 4.1 The influence of 5%VL squat on the PAPE effect in long jumpers

Jump height, PPO, and takeoff momentum are key indicators for evaluating vertical jump performance (11, 53). The present study demonstrated that 4 min after completing squats with a 5%VL threshold, participants exhibited significant increases in CMJ height, PPO, and momentum compared with baseline values. These findings indicate that, under the present experimental conditions, the potentiation effect induced by the conditioning activity outweighed the fatigue generated concurrently (54, 55). This observation is consistent with conclusions reported in recent literature.

Previous evidence suggests that performance progressively declines as training volume increases, implying that greater VL exacerbates fatigue accumulation and may attenuate potentiation effects. González-Badillo et al. (14) reported a strong association (*r*^2^ = 0.83) between VL and the percentage of maximum repetitions completed. Similarly, Weakley et al. (56) observed increases in ratings of perceived exertion (RPE) and blood lactate concentration, accompanied by reductions in immediate jump height under fixed external loads as VL increased, thereby confirming the amplifying effect of higher training volume on fatigue.

A single muscular contraction simultaneously induces both potentiation and fatigue (57), and the ultimate manifestation of PAPE depends on the relative magnitude and temporal interaction of these two processes. Hamada et al. (58) proposed that excessive neural activation may result in intramuscular fatigue mechanisms becoming dominant, thereby diminishing the observable potentiation effect. Therefore, selecting an optimal load that effectively elicits PAPE while minimizing fatigue interference represents a critical practical consideration in training design (10, 11).

PAPE specifically refers to the transient enhancement of muscle force-generating capacity following a prior voluntary contraction, which manifests as improved performance in subsequent explosive tasks. Its mechanisms differ from those of post-activation potentiation (PAP) and remain incompletely understood, but likely involve longer-lasting physiological processes. These may include increases in muscle temperature, alterations in blood flow distribution and tissue hydration, elevated muscle activation levels partially influenced by motivational factors, increased plasma catecholamine concentrations, and enhanced excitability of high-threshold motor units (59, 60). Such effects may persist for up to 20 min after the conditioning stimulus, although the precise temporal dynamics require further empirical clarification.

In summary, the findings of this study support the use of a squat protocol with a 5%VL threshold as an effective strategy for enhancing lower-limb power performance in long jumpers. Compared with higher-volume protocols, this approach requires fewer repetitions, allowing neuromuscular potentiation to predominate over fatigue. This may improve training efficiency and potentially reduce injury risk by limiting excessive fatigue accumulation.

### 4.2 The effect of acute BRJ intake on PAPE performance

Previous research suggests that BRJ supplementation may enhance performance in sports characterized by intermittent high-intensity efforts, such as soccer, rugby, and hockey (61–63). Conversely, no significant performance benefits have been reported in other sports, including basketball (64). To date, evidence regarding its effects in long jumpers is limited. Therefore, examining its impact in this population is important for clarifying its practical supplementation value. The present study demonstrated that ingestion of 100 ml of BRJ did not significantly improve CMJ height, PPO, or momentum compared with placebo or control conditions. In other words, acute BRJ supplementation did not produce a clear enhancement of the PAPE effect in male long jumpers. Consequently, our primary hypothesis that acute dietary nitrate supplementation would significantly potentiate the PAPE response induced by the conditioning activity was not supported. Notably, the effect sizes for CMJ performance improvements did not favor the BRJ condition (Table 4). In fact, the largest effect sizes were generally observed in the 5%VL and placebo conditions. For example, at the 4-min post-conditioning time point, Cohen's d for CMJ height was 0.661 in the BRJ + 5%VL group, compared with 0.725 in the 5%VL group and 0.693 in the placebo group. These findings support the interpretation that the observed potentiation effects were primarily driven by the squat conditioning protocol rather than nitrate supplementation. This outcome aligns with prior research in basketball athletes (64).

Existing literature suggests that BRJ supplementation can improve endurance performance across various sports (65), potentially through mechanisms involving vasodilation, enhanced muscle blood flow (3, 66), and reduced oxygen cost during submaximal exercise (22). Recent findings in combat sports further suggest that BRJ supplementation improves handgrip strength and forearm muscle oxygen saturation, although it does not appear to augment maximal voluntary contraction (67). However, in the context of explosive power-dominant performance, as examined in this study, acute BRJ supplementation did not significantly augment PAPE outcomes. This contrasts with findings reported by Marechal et al. (68), who observed improvements in muscle velocity and power during high-speed isokinetic knee extension (360°/s) in healthy adults. A notable distinction among these studies lies in the administered nitrate dose, which ranged from 6.4 mmol to 11.2 mmol (69). A lower nitrate dose may be insufficient to adequately activate the cyclic guanosine monophosphate (cGMP) pathway, thereby limiting its potential effects on muscle contractile properties.

Furthermore, CMJ-like movements are frequently performed during long jump training and competition (e.g., bounding and hopping drills). Previous research has shown that supplementation with a relatively low dose of BRJ (70 ml) resulted in only a modest (2.3%) and statistically non-significant increase in jump height (70), which aligns with the present findings (*P* = 0.182). Differences between studies may also be attributable to participant characteristics. The present investigation involved trained male long jumpers, whereas other studies included generally active young males (70). Additionally, some literature reports improvements in peak power during cycling-based resistance tests following BRJ supplementation (31, 70–73), yet similarly failed to detect enhancement in CMJ performance (70).

### 4.3 The effect of acute BRJ intake on squat repetition performance at 5% VL

Previous studies suggest that acute BRJ supplementation may positively influence certain resistance exercise performances, and chronic supplementation protocols have been shown to increase repetition capacity (74, 75). However, the effect of acute BRJ supplementation on repetition performance during squats performed under a fixed VL threshold has not been previously reported. Therefore, in addition to examining PAPE outcomes, the present study investigated the influence of acute BRJ intake on the number of repetitions completed at 85%1RM under a 5%VL condition.

The results demonstrated that acute BRJ supplementation significantly increased the number of squat repetitions completed. Although the present data do not allow definitive elucidation of the underlying physiological mechanisms, this finding provides novel empirical evidence supporting the application of BRJ in enhancing resistance training performance.

A recent meta-analysis on dietary nitrate supplementation in resistance-type explosive performance reported that among 10 included studies, six employed acute supplementation 2–3 h before exercise and four utilized multi-day protocols (76–78). Four studies observed significant improvements, including increases in peak power during flywheel squats (12%−22%) (79), isokinetic knee flexion (2%) (77), isokinetic knee extension (4%) (31), and free-weight bench press (19%) (80), with a 7% increase in mean concentric velocity also reported in the latter (80). Notably, three of these four positive studies used acute supplementation protocols (31, 79, 80). This pattern suggests that acute nitrate supplementation may have distinct potential in resistance exercise contexts.

The present finding that acute BRJ supplementation enhanced dynamic resistance performance (85%1RM squat repetitions) is consistent with previous reports demonstrating improvements in peak isometric force and force output following nitrate supplementation (74). Although physiological mechanisms were not directly assessed, several theoretical explanations may be proposed (27). First, improved muscular contractile efficiency may arise from cellular-level adaptations. Animal studies suggest that nitrate supplementation enhances calcium sensitivity and force production in fast-twitch fibers (21). In humans, acute supplementation may increase cGMP via nitric oxide-soluble guanylate cyclase (NO-sGC) signaling, activating protein kinase G (PKG) to enhance calcium handling and myofilament calcium sensitivity, thereby optimizing cross-bridge cycling and facilitating more efficient and explosive contractions.

Second, modulation of neuromuscular function may contribute. Multi-day BRJ supplementation has been associated with altered motor unit discharge characteristics and increased electromyographic amplitude (81). Acute supplementation may similarly enhance neural drive efficiency by optimizing motor unit recruitment (22), increasing firing frequency, or improving synchronization, thereby enabling greater force production per unit time. Moreover, BRJ supplementation has been reported to reduce metabolic cost within the adenosine triphosphate–phosphocreatine (ATP–PCr) system and improve contraction economy (38). Enhanced metabolic efficiency may delay fatigue during high-intensity resistance exercise, which could manifest as an increased number of repetitions.

Finally, selective hemodynamic adaptations may play a role. Evidence indicates that dietary nitrate supplementation may preferentially enhance blood flow to fast-twitch muscle fibers (23). Given the high metabolic demands of these fibers during high-intensity, multi-repetition efforts, improved substrate delivery and metabolite clearance may support sustained force output (82, 83). Collectively, the ergogenic effect of acute BRJ supplementation on resistance exercise performance likely reflects a combination of molecular, neuromuscular, and hemodynamic mechanisms.

### 4.4 Limitations

Although this study provides preliminary evidence regarding the effects of BRJ supplementation on resistance exercise performance, several limitations should be acknowledged. First, plasma nitrite concentrations were not measured; therefore, the specific concentration threshold associated with performance enhancement could not be determined. Nevertheless, previous studies using similar supplementation protocols have consistently demonstrated significant elevations in circulating nitrite levels (84, 85), providing indirect support for the proposed mechanisms. Second, the participant sample included only male long jumpers, limiting the generalizability of findings to female athletes. Potential sex-specific responses to nitrate supplementation warrant further investigation. Third, the present study focused exclusively on acute supplementation and did not examine the cumulative effects of chronic intake on neuromuscular adaptations or strength development. Future research should explore both acute and chronic supplementation strategies and directly assess underlying physiological mechanisms. Fourth, the nitrate-depleted placebo was prepared via ion exchange resin but sourced from a different supplier than the active active BRJ supplement. As a result, other bioactive constituents naturally present in beetroot (e.g., polyphenols, vitamins, and additional micronutrients) may not have been fully matched between active and placebo conditions. While dietary nitrate is widely regarded as the principal driver of ergogenic effects, emerging evidence suggests potential synergistic interactions with other beetroot-derived compounds. Nevertheless, the scarcity of commercially available nitrate-depleted BRJ products underscores the validity of our placebo selection approach. In summary, acute BRJ supplementation significantly increased the total number of repetitions performed during free-weight squats at 85%1RM, although it did not enhance CMJ-based PAPE outcomes. Further studies are required to clarify the mechanistic basis of these findings and to systematically compare the effects of acute vs. chronic nitrate supplementation on resistance training adaptations.

## 5 Conclusions

The primary findings of this study are as follows. First, a squat protocol employing a 5%VL threshold effectively induced a PAPE response in long jumpers. However, acute BRJ supplementation did not further enhance single explosive jump performance. Notably, BRJ supplementation was associated with an increased number of squat repetitions performed at 85%1RM, suggesting a potential benefit for strength-endurance capacity. These results indicate that low-volume activation protocols can be used to acutely improve explosive performance, while acute BRJ supplementation may support the maintenance of training volume. Overall, these findings provide practical, cautiously interpreted insights for the short-term training and performance strategies of long jumpers.

## Data Availability

The datasets generated and/or analyzed during the current study are not publicly available because they are part of an ongoing research project. Public release of the data at this stage could affect the integrity of the continuing research. Data will be made available by the corresponding author upon reasonable request once the project has been completed, subject to applicable ethical and institutional requirements.
